# A Microfluidic Gradient Platform for High-Throughput Evaluation of Blue-Light-Induced Oxidative Stress and Antioxidant Protection in Retinal Pigment Epithelial Cells

**DOI:** 10.3390/bios16090516

**Published:** 2026-09-12

**Authors:** Hon-Man-Herman Tam, Sheng-Yen Wang, Yung-Shin Sun, Kai-Yin Lo

**Affiliations:** 1 Department of Agricultural Chemistry, National Taiwan University, Taipei City 10617, Taiwan; d13623005@ntu.edu.tw; 2 Department of Physics, Fu-Jen Catholic University, New Taipei City 24205, Taiwan

**Keywords:** retinal pigment epithelium, blue light, reactive oxygen species, microfluidics, antioxidants

## Abstract

The retinal pigment epithelium (RPE) is a monolayer of cells located between retinal photoreceptors and the choroid, playing a critical role in maintaining visual function by protecting the retina and supporting photoreceptor metabolism. Damage to RPE cells can lead to visual disorders, including macular degeneration. Chronic exposure to high-energy blue light has been shown to elevate intracellular reactive oxygen species (ROS) in RPE cells, causing oxidative stress and cellular damage. In this study, a microfluidic platform incorporating a gradient-generating structure was developed to establish controllable and stable gradients of blue light intensity and chemical concentrations. This platform was used to investigate the effects of varying blue light intensities and antioxidant concentrations on oxidative stress in human RPE cells ARPE-19. Cells cultured within the microfluidic channels were exposed to different blue light intensities in combination with chemical treatments. Results demonstrated that ROS production increased with higher blue light intensity, whereas higher antioxidant concentrations effectively reduced ROS accumulation, supporting the ability of these antioxidants to attenuate blue-light-induced intracellular oxidative stress. The present microfluidic device enables simultaneous evaluation of multiple conditions within a single experiment, reducing reagent consumption and enhancing experimental efficiency. This in vitro microfluidic platform integrates chemical and light gradients to assess retinal oxidative damage and antioxidant effects, offering significant potential for ophthalmic drug screening and investigations of retinal protective mechanisms.

## 1. Introduction

Blue light, typically defined as visible light with wavelengths between 400 and 500 nm, is an integral component of both natural and artificial lighting environments. Sunlight is the primary natural source of blue light, and daytime exposure is essential for regulating circadian rhythms, sleep–wake cycles, and hormone production, including melatonin [1,2]. However, in modern society, artificial sources of blue light have become increasingly prevalent. Light-emitting diode (LED) lighting, fluorescent lamps, and digital devices such as smartphones, tablets, and computers have led to continuous and prolonged exposure, particularly in indoor and nighttime environments. The intensity, proximity, and duration of exposure to these devices often result in higher retinal doses than natural sunlight filtered through the ocular media, raising concerns about potential ocular and systemic health effects. Understanding the physiological consequences of this widespread exposure is therefore critical in the context of contemporary lifestyles.

Excessive or chronic exposure to blue light can induce significant retinal damage through multiple mechanisms [3,4]. Due to its relatively high photon energy, blue light can penetrate the cornea and lens to reach the retinal pigment epithelium (RPE) and photoreceptor cells [5]. A primary mechanism of injury involves the generation of reactive oxygen species (ROS), leading to oxidative stress, mitochondrial dysfunction, and apoptotic cell death [6,7]. Oxidative stress can impair photoreceptor function and disrupt the phagocytosis of photoreceptor outer segments by the RPE, compromising retinal metabolism and protective functions [8,9]. Furthermore, blue light exposure can trigger inflammatory and stress-response pathways within retinal cells, exacerbating tissue injury [4]. Experimental models, including in vitro cell cultures and animal studies, consistently demonstrate that prolonged blue light exposure increases retinal vulnerability, suggesting that cumulative exposure over time may contribute to degenerative changes in the macula and other retinal layers [7]. Retinal damage from blue light has been implicated in several ocular diseases. Age-related macular degeneration (AMD) is the most extensively studied condition, characterized by progressive central vision loss due to macular degeneration [7]. Oxidative stress induced by blue light in the RPE is a key factor accelerating AMD pathogenesis [10]. In addition, chronic exposure is associated with digital eye strain, manifested as visual fatigue, dryness, blurred vision, headaches, and reduced accommodation [11,12]. Blue light may also contribute to cataract formation through photochemical damage to lens proteins, although this relationship requires further investigation [3,13]. These findings highlight that, while blue light is essential for normal physiological processes, excessive or prolonged exposure can become a significant risk factor for both degenerative and functional ocular disorders.

To mitigate blue-light-induced retinal damage, a combination of behavioral, physical, and nutritional strategies is recommended. Limiting screen time, adjusting device brightness and color temperature, and reducing exposure to blue-light-emitting devices during evening hours are effective behavioral approaches. Physical interventions, such as blue-light-filtering lenses and screen protectors, can reduce retinal exposure, although their long-term protective effects remain under study [14,15]. Nutritional strategies have also shown promise in enhancing retinal resilience. Antioxidants, including vitamins C and E, lutein, zeaxanthin, and omega-3 fatty acids, play a critical role in neutralizing reactive oxygen species and protecting retinal cells from oxidative stress [16,17]. Studies indicate that diets rich in these nutrients can improve macular pigment density, reduce oxidative damage, and potentially slow the progression of AMD [18,19].

Traditional in vitro investigations of blue-light-induced effects on RPE cells are predominantly conducted using conventional cell culture dishes or multiwell microplates. In these approaches, RPE cells are typically exposed to uniform blue light illumination under static culture conditions, followed by endpoint assays to evaluate cell viability, oxidative stress, apoptosis, or gene expression [20,21]. For example, by employing microplate-based assays and a compatible reader, Marie et al. determined the precise action spectrum of oxidative stress in A2E (N-retinylidene-N-retinylethanolamine)-loaded RPE cells, revealing that blue-violet light (390–520 nm, 0.11–1.55 mW/cm^2^) exposure triggered pronounced generation of both hydrogen peroxide and superoxide anions [22]. In another study, RPE cells cultured in 96- or 48-well plates and exposed to blue light (465–475 nm, 26 W/m^2^) exhibited reduced cell viability, mitochondrial depolarization, increased reactive oxygen species production, and upregulated expression of cell-death-associated proteins [23]. Exposure of A2E to blue light (430 nm, 50 mW/cm^2^) triggered ferroptosis in RPE cells cultured in microplates, accompanied by elevated intracellular ferrous ion levels [24]. While these conventional methods are well established and technically straightforward, they present several important limitations. First, static cultures fail to recapitulate the dynamic microenvironment of the retina, including physiological fluid exchange, nutrient gradients, and waste removal, which can significantly influence cellular responses to photo-induced stress [25,26]. Second, light exposure in dishes or microplates often lacks precise spatial and temporal control, leading to non-uniform irradiation and potential variability between experiments. In addition, these platforms provide limited capability to generate or maintain concentration gradients of oxygen, metabolites, or reactive oxygen species, which are known to play critical roles in blue-light-mediated RPE damage. Together, these drawbacks limit the physiological relevance and mechanistic insight obtainable from traditional dish- or microplate-based models when studying blue-light-induced RPE dysfunction.

Microfluidic devices offer an effective strategy to overcome many of the above-mentioned limitations. By enabling continuous perfusion, microfluidic platforms better mimic the dynamic retinal microenvironment, providing controlled nutrient supply, waste removal, and physiologically relevant shear stress [27,28]. Importantly, microfluidic systems allow precise spatial and temporal control of light exposure and cellular microenvironments, facilitating uniform and reproducible irradiation conditions [29,30]. The small length scales of microchannels also enable the generation and maintenance of well-defined gradients of oxygen, chemicals, or metabolites, which are critical for cell-related studies [31,32]. Moreover, microfluidic devices are compatible with real-time, noninvasive imaging and on-chip sensing, permitting continuous monitoring of cellular responses and revealing cell-to-cell heterogeneity that is obscured in bulk assays [33,34]. For example, Mishra et al. developed a convenient diffusion-based microfluidic system that replicates the geometry and scale of the mouse and human retina, enabling the investigation of concentration-dependent retinal progenitor cell migration to improve existing therapies and to facilitate the development of novel migration-targeted treatments [35]. An alternative study reported the development of a multi-compartment microfluidic device that permits precise spatial segregation and coculture of critical retinal cell types, enabling quantitative assessment of cell–cell interactions across retinal layers [36]. Collectively, microfluidic-based models provide a more physiologically relevant, controllable, and informative platform for investigating the mechanisms of blue-light-induced retinal damage.

Another issue that requires attention is that most previous studies use a single light dose per experiment, leading to low efficiency and limited experimental throughput. The power of the light source should be adjustable to allow variation in light dose. To overcome this limitation, we developed a microfluidic chip capable of generating five distinct blue light doses within a single experimental run. Using a Christmas-tree-shaped microfluidic gradient generator [37,38], we established five well-defined and stable dye concentrations, which were then used to absorb blue light and produce the corresponding light doses. A similar approach was employed in our previous studies. For example, Huang et al. generated five distinct concentrations of blue dye within a single microfluidic chip to investigate the effects of ultraviolet (UV) B radiation on fibroblasts [29]. Lin et al. designed and fabricated a microfluidic chip in which a concentration gradient was first established and then illuminated with a blue LED to generate a blue light gradient, enabling simultaneous cell culture and the observation of cell migration in response to the light stimulus [30]. In this study, a similar design was used to create three different chemical concentrations in cell culture areas to evaluate the effects of antioxidants on the generation of ROS in human retinal pigment epithelium ARPE-19 cells. RPE cells cultured within microchannels were simultaneously subjected to spatially varying blue light exposure and graded antioxidant concentrations. The results revealed a clear light dose-dependent increase in intracellular ROS levels, while progressively higher antioxidant concentrations markedly attenuated ROS accumulation, demonstrating their protective efficacy against blue-light-induced oxidative damage. By enabling parallel testing of multiple experimental conditions within a single device, this microfluidic approach minimizes reagent usage and significantly improves experimental throughput and reproducibility. The integration of optical and chemical gradients within this in vitro platform provides a powerful and versatile tool for mechanistic studies of retinal photo-oxidative injury and holds strong promise for efficient screening of therapeutic agents aimed at retinal protection.

## 2. Materials and Methods

### 2.1. Chip Design and Fabrication

**Figure 1:**
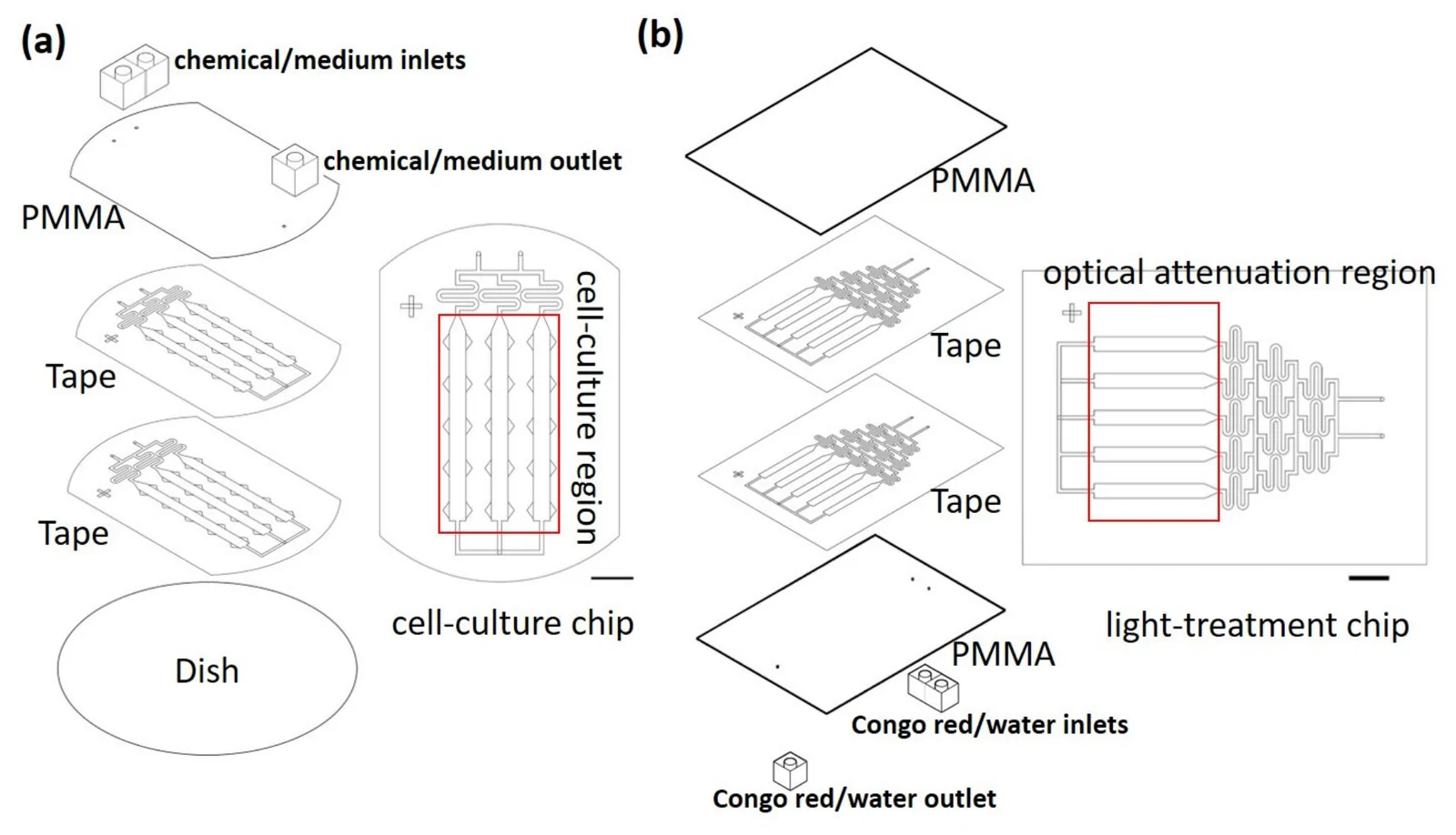
(**a**) Design of the cell culture chip. From top to bottom: 2 mm PMMA layer with adaptors, 260 μm double-sided tape (culture area), 60 μm double-sided tape (culture area), and cut Petri dish (culture surface). Scale bar = 1 cm. (**b**) Design of the light treatment chip. From top to bottom: 2 mm PMMA layer, 260 μm double-sided tape (shielding area), 60 μm double-sided tape (shielding area), and 2 mm PMMA layer with adaptors. The light-treatment chip is positioned beneath the cell-culture chip during blue light exposure, allowing the Congo red concentration gradient to generate a corresponding spatial gradient of transmitted blue light intensity. Scale bar = 1 cm.

The present microfluidic platform consists of two components: a cell culture chip and a light treatment chip, as illustrated in Figure 1. Device layouts were designed using AutoCAD 2026 (Autodesk, San Francisco, CA, USA) and subsequently transferred to a CO_2_ laser engraving system (MS640D, Ming-Cheng Technics Corp., Nantou, Taiwan) for precise ablation of predefined patterns onto polymethylmethacrylate (PMMA) substrates and double-sided adhesive films (3M^TM^ 8018, 3M, St. Paul, MN, USA). The cell culture chip (see Figure 1a, from top to bottom) consisted of one PMMA sheet (2 mm thickness), two double-sided adhesive tapes with thicknesses of 260 μm and 60 μm, and a cut Petri dish (Thermo Fisher Scientific, Waltham, MA, USA). The top PMMA layer was fabricated with three through-holes that functioned as fluidic inlets and outlets via square adaptors, allowing controlled introduction and removal of solutions. Beneath this layer, two successive double-sided tapes defined the microfluidic channels for cell culture, with a combined channel height of approximately 320 μm. The bottom Petri dish was used to promote improved cell adhesion. In the light treatment chip (see Figure 1b), the bottom PMMA layer contained three through-holes serving as fluidic inlets and outlets. Above this layer, two successive double-sided tapes defined the microfluidic channels of the shielding region. The topmost PMMA sheet served as the enclosed layer. To evaluate the biocompatibility of the device materials, cytotoxicity assays were performed using PMMA substrates. No statistically significant reduction in cell viability was observed, confirming that the PMMA-based microfluidic platform is suitable for cell-based studies [39].

### 2.2. Calculation and Simulation of Concentrations and Absorbance

**Figure 2:**
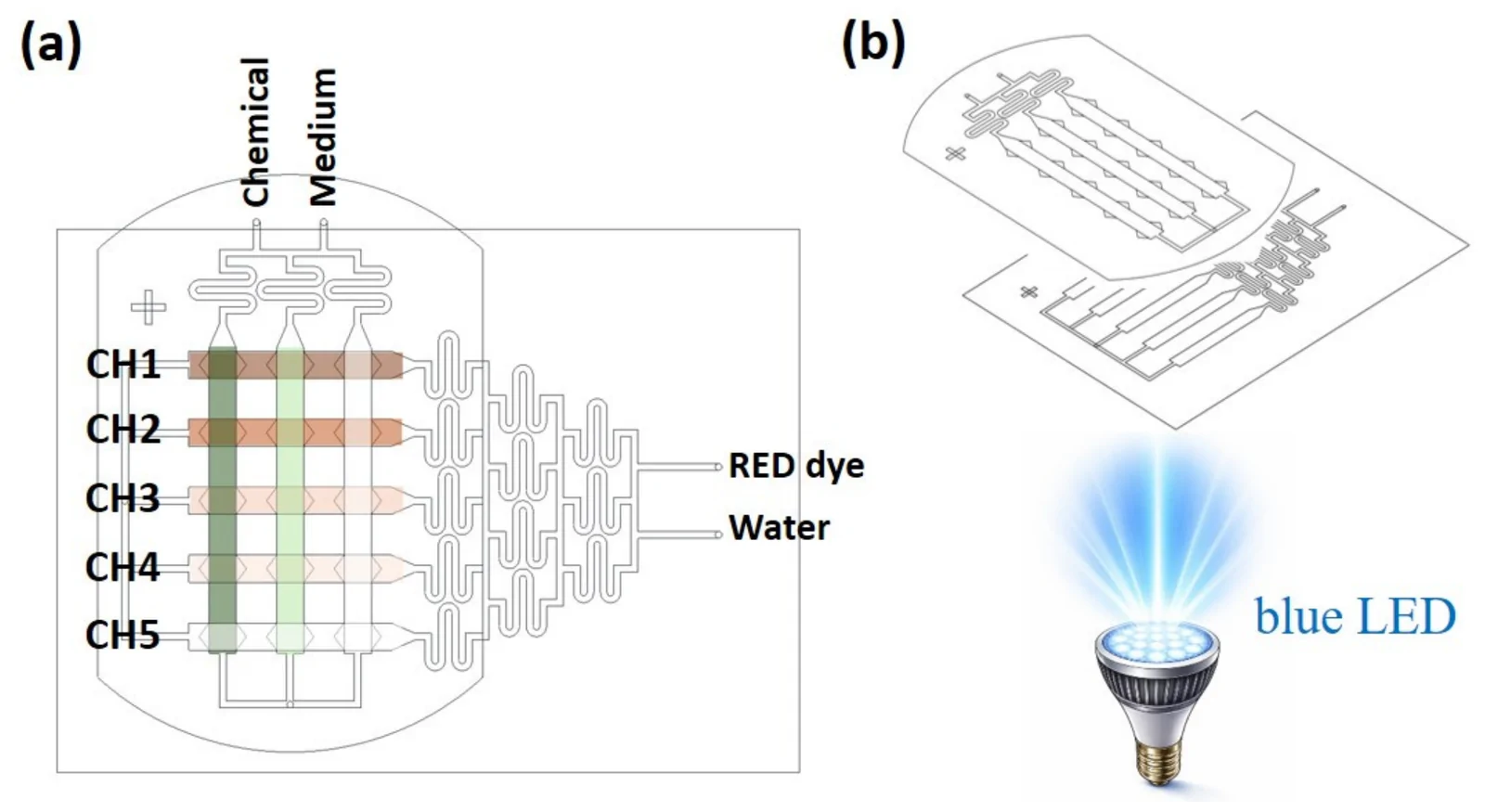
(**a**) The microfluidic platform used in the study. (**b**) The experimental setup of this study. The cell-seeded culture chip is stacked above the light-treatment chip, with blue LED light delivering upward from the bottom.

As shown in Figure 1 and Figure 2, the cell-culture chip and light-treatment chip function as two physically separate but optically coupled modules: the former establishes the antioxidant concentration gradient and provides the cell-culture regions, whereas the latter generates the Congo red concentration gradient used to control the transmitted blue light intensity. A Christmas-tree-type microchannel network was used to simultaneously establish concentration gradients of chemicals within the cell culture chip and red dye within the light treatment chip [37,38]. It is a passive structure designed to generate stable and reproducible concentration gradients under laminar flow conditions. The network consisted of a series of symmetric, bifurcating microchannels that repeatedly split and recombined two inlet streams with different solute concentrations. Two solutions, one containing the target solute at the maximum concentration and the other containing solute-free medium, were introduced into separate inlets at identical volumetric flow rates. At each branching level, the inlet streams were evenly divided and recombined in defined ratios, enabling progressive dilution and diffusive mixing along the channel length. Due to the low Reynolds number in the microchannels, mixing occurred predominantly by molecular diffusion across the fluid interfaces. After multiple stages of splitting and recombination, the network produced a set of outlet channels, each delivering a distinct and well-defined solute concentration. As shown in Figure 2a, in the cell culture chip, when two solutions with relative concentrations of 1 and 0 were introduced through the left (Chemical) and right (Medium) inlets, respectively, three distinct relative concentrations of 0, 0.5, and 1 were generated in the three cell culture regions. Following the same principle, in the light treatment chip, introducing two inlet streams with relative concentrations of 1 and 0 through the top (RED dye) and bottom (Water) inlets resulted in relative concentrations of 0, 0.125, 0.5, 0.875, and 1 in the five shielding areas. The present microfluidic platform enables the simultaneous detection of 15 combined chemical-light stimuli.

In the present study, Congo red (Sigma, Ronkonkoma, NY, USA), an organic, water-soluble dye, was employed as a blue-light-absorbing agent at a concentration of 2.5 mg/mL in double-distilled water (ddH_2_O). The light-treatment chip is capable of generating five distinct Congo red concentrations: 0, 0.3125, 1.25, 2.1875, and 2.5 mg/mL. To characterize the resulting concentration distribution, numerical simulations of solute transport within the microfluidic chip were conducted using COMSOL 6.4 Multiphysics (COMSOL, Burlington, MA, USA). The “Laminar Flow” and “Transport of Dilute Species” modules were employed to simulate fluid dynamics and solute transport within the microfluidic device. At the inlet boundaries, a velocity condition with normal inflow was applied, while the outlet boundary was defined using a pressure condition set to 101,300 Pa, with normal outflow and backflow suppression enabled. The diffusion coefficient of Congo red in ddH_2_O at room temperature was specified as 1 × 10^−10^ m^2^/s [40]. The flow velocity was set to 0.03125 m/s for both inlets. The Congo red concentration at one of the inlets was set to 3.59 mol/m^3^ (2.5 mg/mL), whereas a zero concentration was assigned to the other.

The attenuation of incident blue light by Congo red was analyzed using Beer–Lambert law, which describes the relationship between absorbance (*A*), absorption coefficient (*α*), optical path length (*x*), and solute concentration (*c*) as *A* = *αxc*. Absorbance is also related to the transmittance (*T*) of light through the material according to *A* = −log*T*. Using these two equations, the absorbance (*A*) can be quantitatively related to the concentration (*c*) by measuring the transmitted illuminance beneath the shielding region and normalizing the transmittance to unity for the 0 mg/mL Congo red, in which the incident light is fully transmitted in the absence of a blue-light-absorbing agent.

### 2.3. Cell Culture and Chemicals

Owing to their human origin and reproducible growth characteristics, ARPE-19 cells are a RPE cell line widely used as an in vitro model for studies of retinal physiology, oxidative stress, light-induced retinal injury, and drug or antioxidant responses, providing a relevant and reliable experimental platform for retinal research. These cells exhibit epithelial morphology, form a confluent monolayer under standard culture conditions, and express characteristic RPE markers when appropriately maintained. The cell line was obtained from the Bioresource Collection and Research Center (BCRC, Hsinchu City, Taiwan). Cells were cultured in Dulbecco’s modified Eagle medium/F12 (DMEM/F12, Gibco, Waltham, MA, USA) supplemented with 10% fetal bovine serum (FBS, Sigma, Ronkonkoma, NY, USA), 2.5 mM L-glutamine (Thermo Fisher Scientific, Waltham, MA, USA), 0.5 mM sodium pyruvate (Gibco, Waltham, MA, USA), and 100 U/mL Penicillin-Streptomycin (Gibco, Waltham, MA, USA). Cell cultures were maintained in tissue culture-treated polystyrene dishes (Corning, Corning, NY, USA) at 37 °C in a humidified atmosphere containing 5% CO_2_. Cells were grown to approximately 90% confluence prior to seeding into the microfluidic chip. Two commonly available antioxidants, vitamin C (L-Ascorbic acid, Merck, Rahway, NJ, USA) and vitamin E (α-tocopherol, Sigma, Ronkonkoma, NY, USA), as well as uric acid (Cyrusbioscience, New Taipei City, Taiwan), one of the most abundant endogenous antioxidants in the body, were dissolved in culture medium at the desired concentrations.

### 2.4. Experimental Procedure

#### 2.4.1. Chip Assembly and Cell Preparation

Two microfluidic chips (see Figure 1) were assembled under sterile conditions in a laminar flow hood. For each chip, the inlets were connected to two syringes driven by syringe pumps (NE-300, New Era, Buffalo, NY, USA), and the outlet was connected to another syringe for waste collection. Before seeding cells into the cell culture chip, the microfluidic chip was coated with poly-L-lysine (0.1 mg/mL; Sigma, Ronkonkoma, NY, USA) for 30 min and then rinsed with 1× phosphate-buffered saline (PBS). ARPE-19 cells (~1 × 10^6^ cells) suspended in 1 mL of culture medium were subsequently introduced into the culture regions through the two inlets and incubated at 37 °C in a humidified atmosphere containing 5% CO_2_ for 3 h to allow cell attachment. As illustrated in Figure 2b, for light stimulation experiments, the cell-seeded culture chip was mounted onto the light-treatment module, and blue LED light was applied from below.

#### 2.4.2. Blue Light Treatments

After 3 h of incubation under a continuous flow of fresh culture medium at 10 μL/min, the two inlets of the culture chip were connected to culture medium and a chemical solution at a specified concentration, respectively, both injected at a flow rate of 10 μL/min. Simultaneously, syringes containing water and 2.5 mg/mL Congo red were connected to the two inlets of the treatment chip and driven by syringe pumps at 10 μL/min for at least 30 min to establish a stable concentration profile. A blue LED light (PAR30 E27 13W 450–460 nm) was positioned approximately 35 cm below the microfluidic platform (see Figure 2b). During the treatment duration of 2 h, both the water and dye solutions were continuously perfused through the chip to maintain uniform Congo red concentrations within the shielding regions. Blue light illuminance beneath each shielding area was measured using a lux meter (DX-200, INS Enterprise Co., New Taipei City, Taiwan).

#### 2.4.3. Cell Viability Assay

A total of 4000 cells were seeded per well in 96-well plates and cultured overnight. The next day, the antioxidants were diluted in growth medium, and 200 μL of the resulting solution was added to each well. The designated dark-control wells were wrapped in aluminum foil, and the plates were exposed to blue light at an illuminance of 2500 lux for 2 h. After light exposure, the medium was removed, and the cells were gently washed with 400 μL of PBS. Subsequently, 150 μL of 5 mg/mL MTT solution (Sigma, Ronkonkoma, NY, USA) was added to each well, and the plates were incubated in the dark for 2 h. The MTT solution was then carefully removed from each well using a pipette, followed by the addition of 150 μL of dimethyl sulfoxide (DMSO) to dissolve the purple formazan crystals. Absorbance was measured at 540 nm using a SpectraMax^®^ iD3 Multi-Mode Microplate Reader (Molecular Devices, San Jose, CA, USA).

#### 2.4.4. ROS Quantification

Intracellular ROS levels were quantitatively assessed using the fluorescence-based ROS indicator 2′,7′-dichlorodihydrofluorescein diacetate (H_2_DCFDA, Sigma, Ronkonkoma, NY, USA). This probe exhibits excitation and emission maxima at approximately 492–495 nm and 517–527 nm, respectively. Following blue light treatment for 2 h, the cell-culture chip was detached from the light-treatment chip. A 40 μM H_2_DCFDA solution prepared in DMEM (no phenol red) was continuously introduced into the culture chip for 30 min at a flow rate of 10 μL/min. The chip was subsequently flushed with fresh DMEM (no phenol red) at a flow rate of 100 μL/min for an additional 5 min to remove excess dye. Bright-field and fluorescence images of cells cultured in different areas were acquired using an inverted fluorescence microscope (FI40C, ESPA SYSTEMS Co., Hsinchu City, Taiwan) equipped with a digital camera (EOS 60D, Canon, Tokyo, Japan).

### 2.5. Data Analysis

Fluorescence images were analyzed using ImageJ 1.54p software (National Institutes of Health, Bethesda, MD, USA). Individual cells were manually delineated using polygonal regions of interest, and the mean fluorescence intensity within each region was measured. Background fluorescence was subsequently subtracted to obtain the corrected signal intensity. For each experimental condition (one concentration under one light illuminance), approximately 10 cells were analyzed. Data are presented as box plots showing the median, interquartile range, and data dispersion, with whiskers extending to 1.5 times the interquartile range and individual points indicating outliers.

## 3. Results

### 3.1. Results on Calculation and Simulation of Concentrations and Absorbance

**Figure 3:**
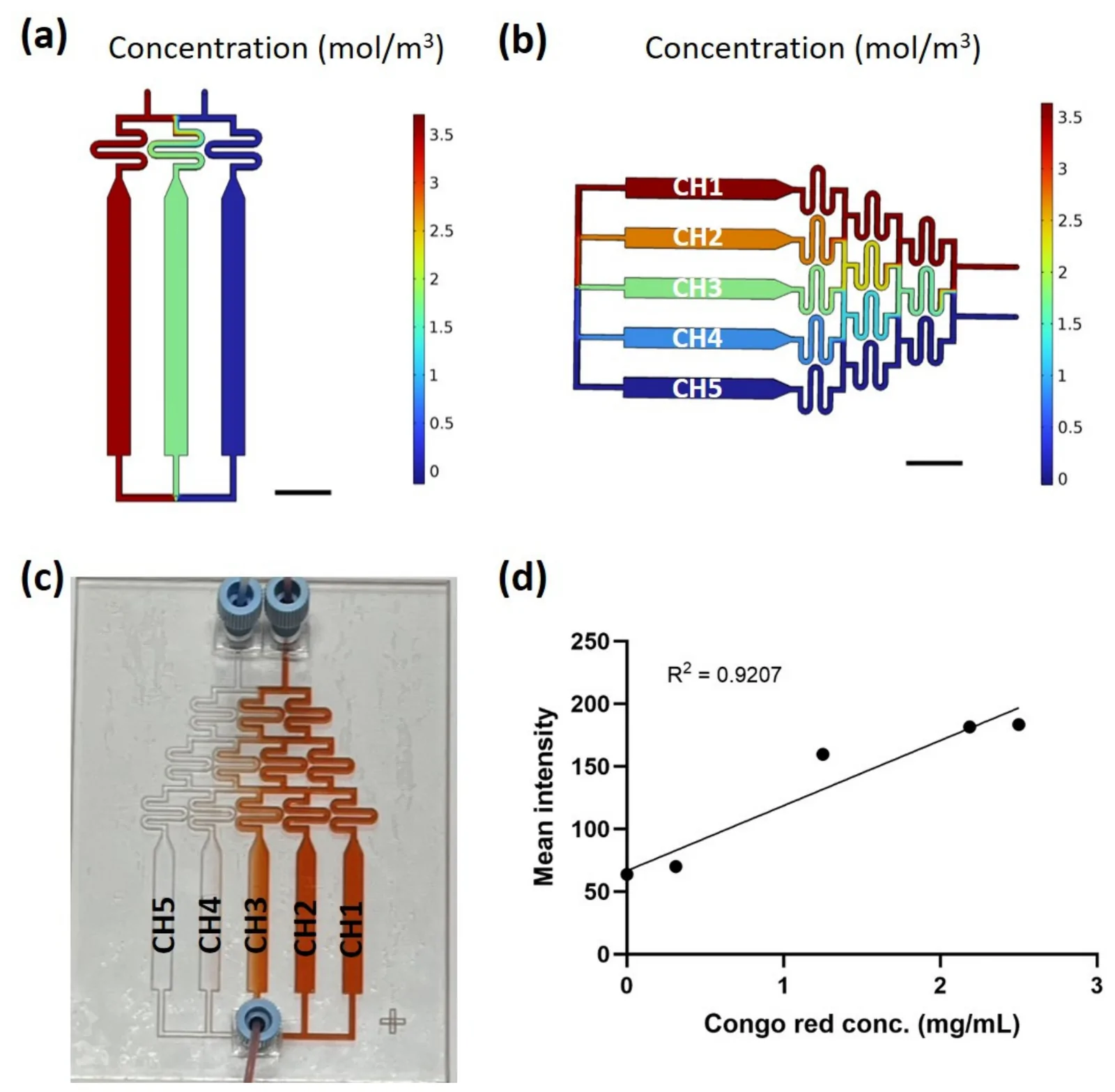
Numerical simulation of Congo red concentration inside (**a**) the cell culture chip and (**b**) the light treatment chip. In both chips, the concentrations in the two inlets are 3.5 and 0 mol/m^3^, respectively. (**c**) Image of the light treatment chip with 0 and 2.5 mg/mL of Congo red continuously flowing into the two inlets. (**d**) Measured intensities (*y*-axis) plotted against calculated concentrations (*x*-axis). N = 3 in (**d**).

Figure 3a and Figure 3b present numerical simulations of the Congo red concentration distributions within the cell culture chip and the light treatment chip, respectively. In both chips, the concentrations at the two inlets were set to 0 and 3.5 mol/m^3^. As shown, the simulated concentrations closely matched the theoretically calculated values of 0, 1.75, and 3.5 mol/m^3^ in the culture chip, and 0, 0.4375, 1.75, 3.0625, and 3.5 mol/m^3^ in the treatment chip. To experimentally validate the concentration distribution within the light treatment chip, Congo red solutions at concentrations of 0 and 2.5 mg/mL were continuously introduced into the two inlets of the chip for 1 h. The entire device was subsequently imaged using an inverted microscope as illustrated in Figure 3c. The mean grayscale intensity within each shielding region was quantified using ImageJ, and the measured values (vertical axis) were plotted against the corresponding calculated concentrations (horizontal axis). A clear linear correlation was observed between the calculated and experimental results, with a coefficient of determination (R^2^) of 0.9207 (see Figure 3d). The error bars are not visible because the standard deviations (SDs; n = 3) are very small.

**Figure 4:**
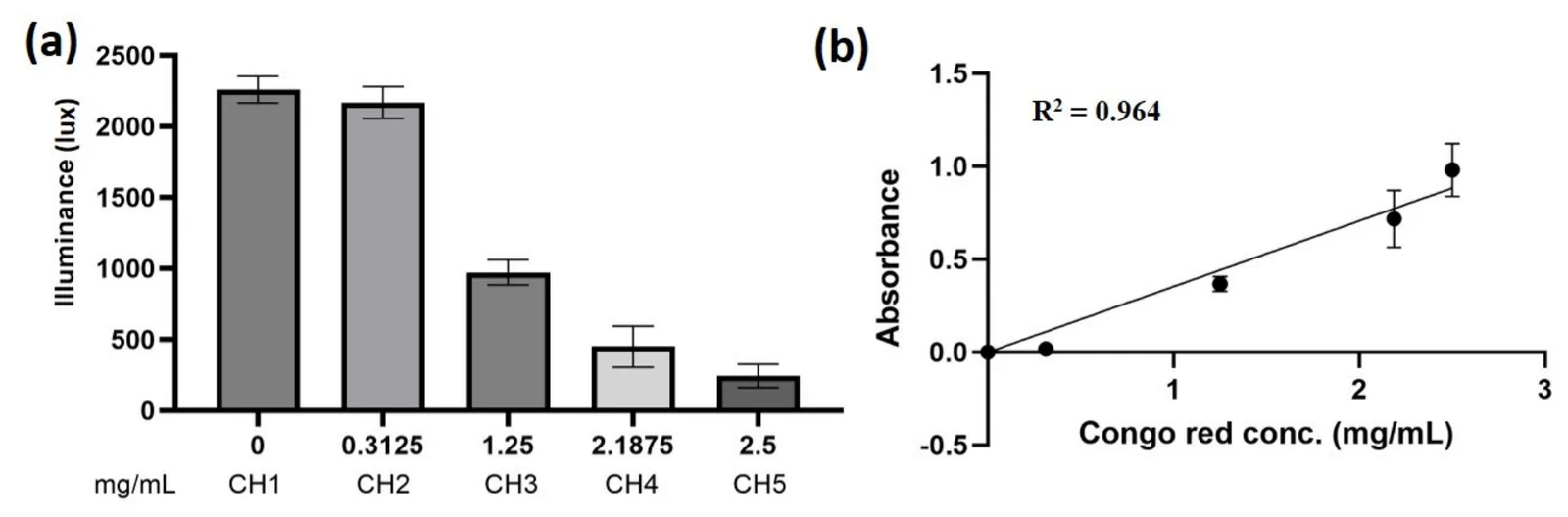
(**a**) Estimated blue light illuminance experienced by cells cultured directly above each of the five shielding areas. (**b**) Relationship between Congo red concentration and blue light illuminance. N = 3 in (**b**).

The blue light illuminance beneath each shielding region was further measured as the incident light passed through the colorant layer. As listed in Table 1, the measured illuminances were approximately 2556 ± 87, 2453 ± 102, 1100 ± 82, 510 ± 134, and 277 ± 76 lux for Congo red concentrations of 0, 0.3125, 1.25, 2.1875, and 2.5 mg/mL, respectively (mean ± SD, n = 3). The transmittance of the Petri dish for blue light was measured to be approximately 88.4%. Accordingly, the estimated illuminances experienced by the cells were 2260 ± 76, 2169 ± 90, 973 ± 73, 451 ± 118, and 245 ± 67 lux under the five shielding conditions, as shown in Figure 4a. This trend indicates that higher dye concentrations more effectively attenuated the transmitted blue light. Then, based on the photometric-to-radiometric conversion for monochromatic blue light (~460 nm) [41], the corresponding irradiance was calculated and is listed in Table 1, along with the energy dose after 2 h of exposure. After normalizing the transmittance to unity under the 0 mg/mL condition, the absorbance values were calculated (see Table 1) and plotted as a function of concentration (see Figure 4b). For example, at a concentration of 1.25 mg/mL, the normalized transmittance (*T*) was calculated as 973/2260 = 0.43, yielding an absorbance (*A*) of −log(*T*) = 0.367. After constraining the absorbance at 0 mg/mL to zero, the resulting linear relationship (*A* = 0.3534*c*) exhibited a high correlation coefficient (R^2^ = 0.964), indicating that the Beer–Lambert law is valid within this concentration range and enabling reliable estimation of absorbance at other concentrations.

### 3.2. Cell Viability Under Blue Light Exposure

To evaluate whether blue light exposure affected cell viability in the presence of different antioxidants, cell viability was assessed under dark conditions and after exposure to blue light at 2500 lux in the presence of vitamin C (VC), vitamin E (VE), or uric acid (UA), as shown in Supplementary Figure S1. Cell viability was normalized to 100% under dark conditions at a concentration of 0 for each antioxidant. As indicated, cell viability remained comparable between the dark and blue light conditions across all tested antioxidant concentrations. For vitamin C, cell viability was approximately 99–114% across the tested concentrations (0, 250, and 500 μM), with no apparent reduction following exposure to 2500-lux blue light. Similarly, in the vitamin E group, cell viability remained comparable between the dark and 2500-lux conditions at concentrations of 0, 125, and 250 μM. Although minor differences were observed between the two illumination conditions at individual concentrations, these changes did not indicate a consistent decrease in cell viability. In the uric acid group, cell viability was also maintained at approximately 98–104% across concentrations of 0, 0.05, and 0.1 mg/mL, with similar values observed under dark and blue light conditions. Overall, blue light exposure at 2500 lux did not adversely affect cell viability in the presence of VC, VE, or UA across the tested concentration ranges. These results indicate that the experimental blue light exposure conditions did not induce a detectable cytotoxic effect on the cells, regardless of the antioxidant treatment.

### 3.3. Effects of Vitamin C on the Generation of ROS in ARPE-19 Cells Under Blue Light Exposure

**Figure 5:**
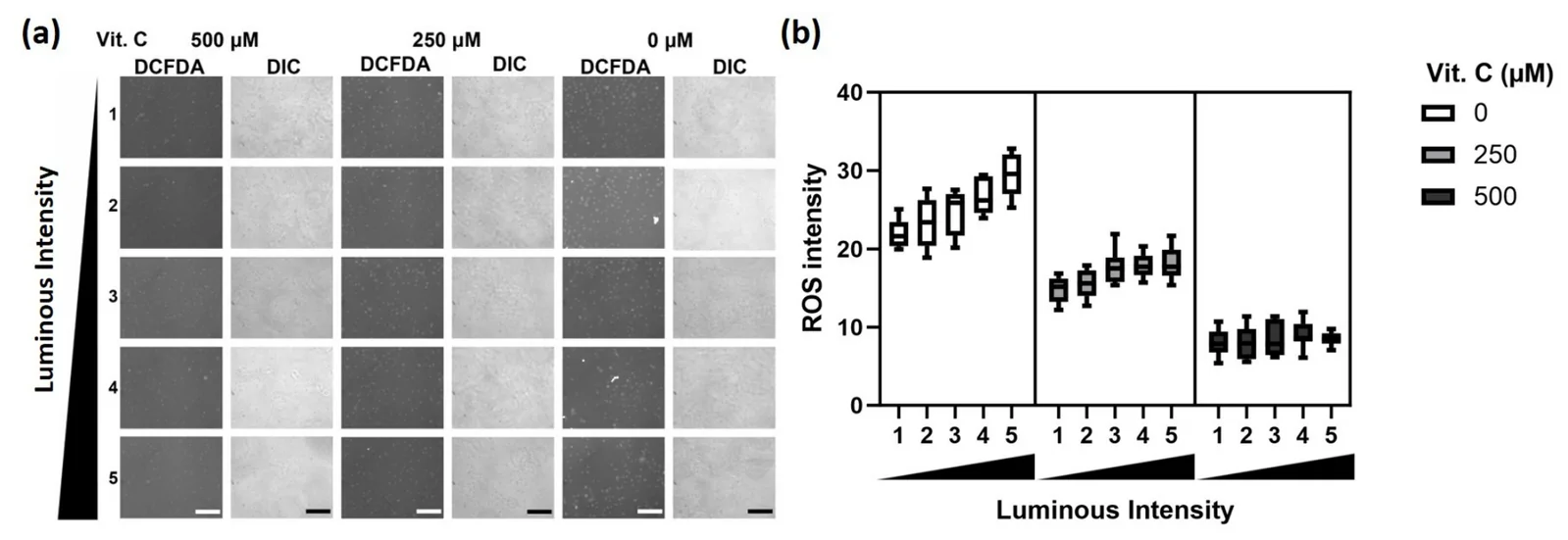
Effects of vitamin C on ROS generation in ARPE-19 cells under different blue light exposure intensities. (**a**) Fluorescence (DCFDA) and differential interference contrast (DIC) images of ARPE-19 cells under different combined vitamin C and blue light exposure conditions. Scale bar = 0.2 mm. (**b**) ROS intensity under different conditions are presented as box plots, where the center line denotes the median, the box represents the interquartile range, and the whiskers extend to the maximum and minimum data points to illustrate data dispersion.

Figure 5 presents the combined effects of blue light illuminance and vitamin C supplementation on intracellular ROS generation in ARPE-19 cells. As shown in Figure 5a, representative fluorescence images obtained using DCFDA staining reveal a progressive increase in intracellular ROS levels with increasing blue light illuminance. This trend was consistently observed across all vitamin C conditions. In contrast, the corresponding differential interference contrast (DIC) images showed no obvious changes in cell morphology or cell distribution, indicating that the observed variations in fluorescence intensity were primarily attributable to changes in ROS generation rather than cell loss or detachment.

Quantitative analysis of ROS intensity in Figure 5b further confirmed the blue light-induced oxidative stress in ARPE-19 cells. In the absence of vitamin C (0 μM), ROS intensity increased monotonically with luminous intensity, rising from 22.03 at 277 lux to 29.40 at 2557 lux. Intermediate luminous intensities (510, 1100, and 2453 lux) produced corresponding ROS intensities of 23.19, 24.61, and 26.67, respectively, demonstrating a clear dose-dependent increase in intracellular ROS generation under blue light exposure. The addition of vitamin C markedly suppressed blue-light-induced ROS production in a concentration-dependent manner. At 250 μM vitamin C, ROS intensities were substantially reduced compared with the untreated group, ranging from 14.73 at 277 lux to 18.07 at 2557 lux, while still exhibiting a modest increase with increasing luminous intensity. Further enhancement of the antioxidant effect was observed at 500 μM vitamin C, where ROS levels were maintained at consistently low values between 8.01 and 9.04 across all tested luminous intensities, indicating effective attenuation of oxidative stress even under high-intensity blue light exposure. A control experiment was performed, indicating that ROS intensity at each vitamin C concentration was comparable between the dark and 300-lux conditions (see Supplementary Figure S2a).

These results demonstrate that blue light exposure induces ROS generation in ARPE-19 cells in a luminous intensity-dependent manner, whereas vitamin C provides robust and dose-dependent protection against oxidative stress. Notably, high-dose vitamin C (500 μM) effectively minimized ROS accumulation across the entire range of blue light intensities, highlighting its strong antioxidant capacity in mitigating blue-light-induced oxidative damage in retinal pigment epithelial cells.

### 3.4. Effects of Vitamin E on the Generation of ROS in ARPE-19 Cells Under Blue Light Exposure

**Figure 6:**
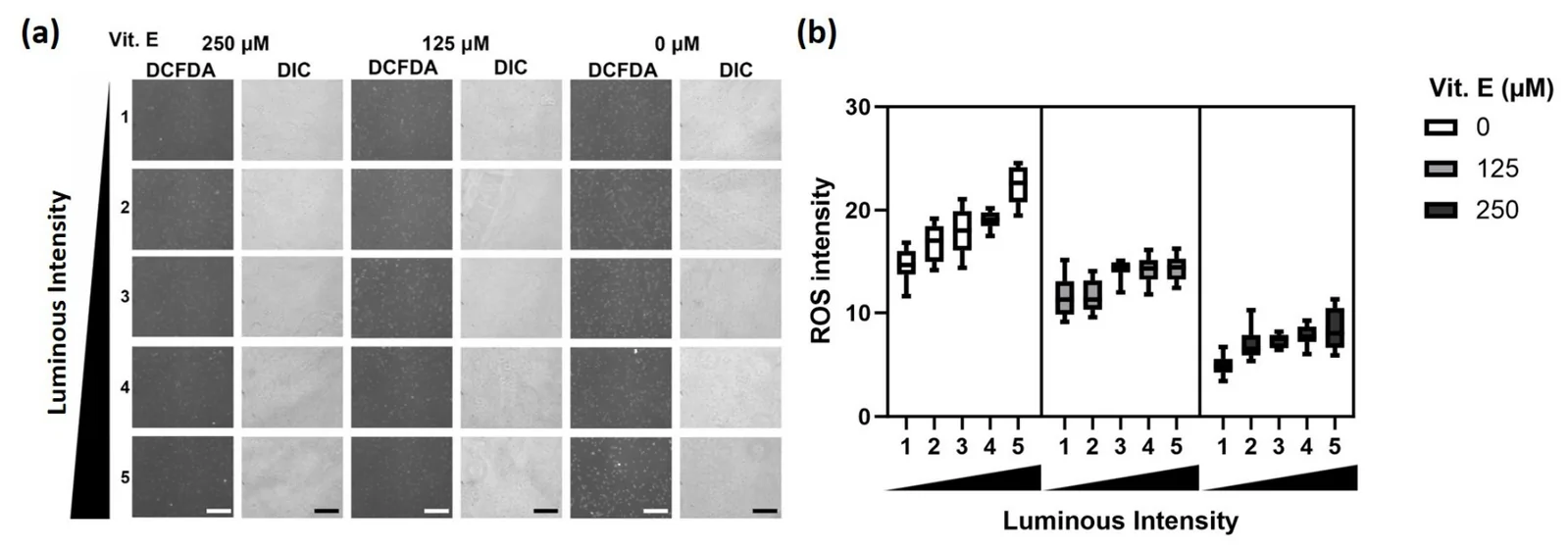
Effects of vitamin E on ROS generation in ARPE-19 cells under different blue light exposure intensities. (**a**) Fluorescence (DCFDA) and differential interference contrast (DIC) images of ARPE-19 cells under different combined vitamin E and blue light exposure conditions. Scale bar = 0.2 mm. (**b**) ROS intensity under different conditions are presented as box plots, where the center line denotes the median, the box represents the interquartile range, and the whiskers extend to the maximum and minimum data points to illustrate data dispersion.

Figure 6 illustrates the effects of blue light exposure and vitamin E supplementation on intracellular ROS generation in ARPE-19 cells. Representative DCFDA fluorescence images in Figure 6a show a clear increase in intracellular ROS levels with increasing luminous intensity under all vitamin E conditions, whereas the corresponding DIC images show no discernible changes in cellular morphology or spatial distribution, indicating that the observed differences in fluorescence intensity predominantly arise from variations in intracellular ROS generation rather than from cell loss, detachment, or gross structural damage.

Quantitative assessment of intracellular ROS levels in Figure 6b revealed a clear dependence of oxidative stress on blue light intensity in ARPE-19 cells. Under vitamin E-free conditions (0 μM), ROS production increased monotonically with rising luminous intensity, escalating from 14.73 at 277 lux to 22.40 at 2557 lux. Intermediate light intensities of 510, 1100, and 2453 lux yielded ROS intensities of 16.78, 17.90, and 19.09, respectively, confirming a pronounced intensity-dependent enhancement of oxidative stress induced by blue light exposure. The presence of vitamin E markedly mitigated blue-light-triggered ROS accumulation across all tested illumination levels. At a concentration of 125 μM, vitamin E significantly lowered ROS intensities relative to the untreated control, with values ranging from 11.55 at 277 lux to 14.34 at 2557 lux, while still maintaining a modest positive correlation with light intensity. In contrast, supplementation with 250 μM vitamin E resulted in robust suppression of ROS generation, maintaining consistently low ROS levels (5.59–7.36) regardless of luminous intensity. Notably, at the highest illumination level (2557 lux), high-dose vitamin E reduced ROS production by approximately 70–75% compared with cells exposed to blue light alone. A control experiment showed that ROS intensity under 300-lux blue light exposure was similar to that measured under dark conditions at all tested vitamin E concentrations (see Supplementary Figure S2b).

These findings demonstrate that blue light induces oxidative stress in ARPE-19 cells in a luminous intensity-dependent manner, whereas vitamin E confers strong, dose-dependent antioxidant protection. In particular, high-dose vitamin E (250 μM) effectively suppressed ROS accumulation even under high-intensity blue light exposure, underscoring its potent antioxidative efficacy in protecting retinal pigment epithelial cells from light-induced oxidative damage.

### 3.5. Effects of Uric Acid on the Generation of ROS in ARPE-19 Cells Under Blue Light Exposure

**Figure 7:**
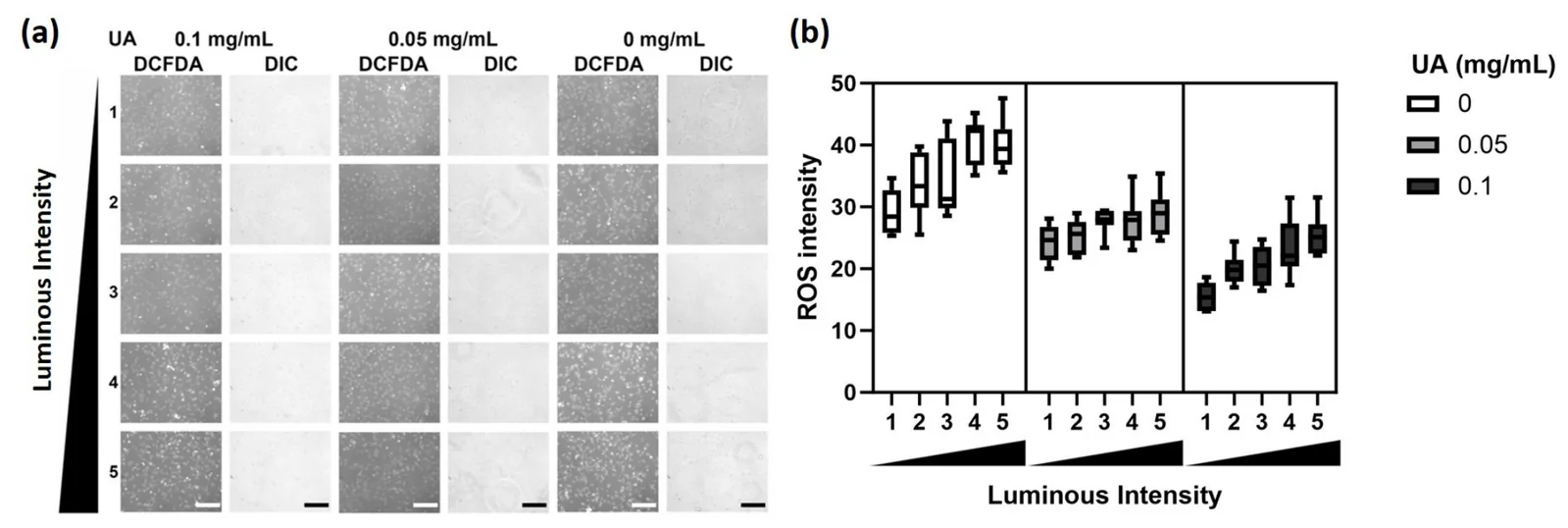
Effects of uric acid on ROS generation in ARPE-19 cells under different blue light exposure intensities. (**a**) Fluorescence (DCFDA) and differential interference contrast (DIC) images of ARPE-19 cells under different combined uric acid and blue light exposure conditions. Scale bar = 0.2 mm. (**b**) ROS intensity under different conditions are presented as box plots showing the median, where the center line denotes the median, the box represents the interquartile range, and the whiskers extend to the maximum and minimum data points to illustrate data dispersion.

Representative fluorescence images of intracellular ROS detected by DCFDA under different blue light intensities and uric acid (UA) concentrations are shown in Figure 7a. In the absence of UA, DCFDA fluorescence gradually increased as the luminous intensity increased from 277 to 2557 lux, indicating enhanced ROS generation under stronger blue light exposure. The fluorescence signals appeared dense and bright at higher intensities, while weaker fluorescence was observed at lower light levels. In contrast, cells treated with UA exhibited visibly reduced fluorescence signals across all light intensities. The reduction was most evident at the higher UA concentration (0.1 mg/mL), where the fluorescence signal remained comparatively weaker even under the highest light intensity. DIC images showed similar cell morphology across conditions, suggesting that the observed differences in fluorescence primarily reflected changes in intracellular ROS levels rather than major morphological alterations.

Quantitative analysis of ROS intensity is summarized in Figure 7b. In the absence of UA, ROS intensity increased with increasing light intensity, rising from 29.4 at 277 lux to 41.1 at 2557 lux. Intermediate intensities of 510, 1100, and 2453 lux produced ROS intensities of 33.6, 34.3, and 40.6, respectively, indicating an overall upward trend in ROS generation with increasing illumination. Addition of UA reduced ROS levels at all light intensities. At 0.05 mg/mL UA, ROS intensities ranged from 24.2 at 277 lux to 29.0 at 2557 lux, representing a reduction of approximately 17–29% compared with the corresponding conditions without UA. A further decrease in ROS generation was observed at 0.1 mg/mL UA. Under this condition, ROS intensities ranged from 15.4 at 277 lux to 25.2 at 2557 lux, corresponding to reductions of approximately 35–48% relative to the UA-free condition. Similar to the results obtained with vitamin C and vitamin E, ROS intensity under 300-lux blue light exposure was comparable to that under dark conditions across the tested UA concentrations (see Supplementary Figure S2c).

Overall, ROS production increased with increasing blue light intensity, whereas UA treatment suppressed ROS generation in a concentration-dependent manner. The higher UA concentration (0.1 mg/mL) consistently produced the lowest ROS levels across all illumination conditions, indicating a stronger protective effect against blue-light-induced oxidative stress.

## 4. Discussion

### 4.1. Congo Red-Mediated Blue Light Absorption

Congo red is a synthetic anionic diazo dye composed of two azo (–N=N–) linkages connecting aromatic rings substituted with sulfonate groups, which confer high water solubility and strong affinity for charged and planar biomolecular structures [42]. From an optical perspective, Congo red exhibits a broad absorption spectrum in the visible range, with a prominent absorption maximum typically centered around 490–500 nm in aqueous solution [43]. As a result, Congo red strongly absorbs blue to blue-green light, leading to efficient attenuation of short-wavelength visible radiation. The absorption profile arises from π–π* electronic transitions within its extended conjugated aromatic system, making Congo red an effective optical filter or light-absorbing agent in applications requiring controlled modulation of blue light intensity [43]. In our previous studies, a blue colorant, Brilliant Blue FCF, was used to attenuate different amounts of UV and blue light to investigate the effects of UV-B dose on NIH/3T3 fibroblasts and the phototactic responses of lung cancer cells, respectively [30,39]. However, this blue dye exhibits a strong absorption maximum at around 628–630 nm, with high absorbance mainly in the 550–650 nm range [44]. In contrast, absorbance in the blue light region (400–500 nm) is relatively weak and arises only from a low-intensity absorption tail. Consequently, attenuation of blue light is limited and required relatively high dye concentrations. These results suggest that relative to Congo red used in the present study, Brilliant Blue FCF is less suitable for precise modulation of blue light intensity.

The results presented in Figure 3 and Figure 4 demonstrate that the proposed microfluidic platform enables precise and predictable modulation of both chemical concentration and transmitted blue light intensity. Numerical simulations confirmed that the gradient-generating architectures in the cell culture and light treatment chips produced well-defined, stepwise concentration profiles that closely matched theoretical predictions, while a clear linear relationship between simulated and experimentally measured Congo red distributions in the treatment chip (R^2^ = 0.9144) verified that diffusion-dominated transport within the designed microchannels is stable, reproducible, and robust. Importantly, the experimentally established Congo red concentration gradient was directly translated into a controllable blue light attenuation gradient, as evidenced by the monotonic decrease in transmitted illuminance with increasing dye concentration. This behavior confirms that spatial modulation of light intensity can be achieved passively through solution-mediated optical filtering, without altering the light source or relying on complex optical components.

In contrast to LED array-based blue light platforms, which require multiple independently controlled light sources, extensive calibration, and precise spatial alignment to generate heterogeneous illumination, the present system achieves continuous light modulation through microfluidic design and inlet concentration control alone [45,46]. LED arrays are additionally constrained by discrete emitter spacing, nonuniform emission profiles, and potential thermal effects, which can introduce variability in delivered light dose. Similarly, photomask- or filter-based approaches typically produce fixed, stepwise illumination patterns and lack flexibility, as modifying light intensity or spatial distribution necessitates physical replacement or redesign of optical elements. By comparison, the proposed platform converts a chemically defined gradient into a predictable optical attenuation profile, enabling smooth, scalable, and programmable light gradients within a single device.

Moreover, the linear relationship observed between absorbance and Congo red concentration (R^2^ = 0.9639) indicates that the Beer–Lambert law remains valid within the investigated range, providing a quantitative framework for predicting light attenuation based solely on dye concentration. This intrinsic linearity allows illumination profiles to be designed a priori through microfluidic architecture and inlet conditions, offering a level of predictability and adaptability that is difficult to achieve with conventional optical filtering strategies. Collectively, these features position the present platform as a versatile and high-precision alternative to existing blue light exposure systems, particularly for studies of light-induced cellular responses such as oxidative stress, phototoxicity, and photobiomodulation, where accurate control of local light dose is essential.

### 4.2. Effects of Blue Light on the Generation of ROS in ARPE-19 Cells

Exposure to blue light has been widely reported to induce excessive intracellular ROS generation in ARPE-19 cells, leading to oxidative stress and subsequent cellular dysfunction. Short-wavelength visible light (approximately 400–470 nm) is readily absorbed by mitochondrial chromophores, including flavins and cytochrome c oxidase, resulting in impaired electron transport and enhanced electron leakage, which promotes superoxide formation and mitochondrial ROS production [47]. For example, compared with the dark control, blue light treatment (465–475 nm, 400 lux, 36 h exposure) reduced the viability of ARPE-19 cells to approximately 50% and caused a substantial increase in ROS generation [48]. Godley et al. found that exposure of non-pigmented human retinal epithelial cells to visible blue light (390–550 nm, 2.8 mW/cm^2^) induced time- and dose-dependent increases in ROS, resulting in reduced mitochondrial respiratory activity and significant mitochondrial DNA damage detectable within 3 h, with partial recovery observed after prolonged exposure due to DNA repair [49]. In another study, blue light exposure (465–475 nm, 800 lux, 3–12 h exposure) significantly reduced ARPE-19 cell viability, depolarized mitochondria, increased ROS, and activated cell-death pathways [23]. Also, blue light treatment (440–445 nm wavelength, 2200 lux, 30 min exposure) caused notable death of ARPE-19 cells primarily through a regulated necrotic pathway, characterized by increased intracellular oxidative stress, loss of mitochondrial membrane potential, release of nuclear proteins, aggregation of cytoplasmic signaling proteins, and disruption of cell membrane integrity, while nuclear membranes remained intact [50]. In cultured ARPE-19 cells exposed to blue light LED (50–1000 lux) for 4 h, cell viability and mitochondrial membrane potential decreased in a dose-dependent manner starting at 50 lux, membrane damage was observed at ≥100 lux, ROS production increased from ≥250 lux, and apoptosis became evident at 1000 lux [51].

In the present study, Figure 5, Figure 6 and Figure 7 clearly demonstrate that blue light treatment induces intracellular ROS generation in ARPE-19 cells in a luminous intensity-dependent manner. As blue light illuminance increased, ROS levels rose progressively, indicating that higher photon flux exacerbates oxidative stress in RPE cells. This observation is consistent with the high-energy nature of short-wavelength visible light, which readily excites intracellular chromophores and photosensitizers, including flavins, porphyrins, and lipofuscin-related compounds, leading to the formation of reactive oxygen intermediates [47]. The absence of pronounced morphological changes in the DIC images suggests that ROS accumulation occurs prior to overt structural damage or cell detachment. This finding highlights oxidative stress as an early cellular response to blue light exposure and supports its use as a sensitive indicator of phototoxicity. Given the central role of RPE cells in maintaining retinal integrity and supporting photoreceptor metabolism, sustained or repeated blue-light-induced ROS generation may compromise cellular function and contribute to the initiation of retinal degenerative processes [52]. These results further reinforce the importance of controlling blue light exposure, particularly under high-intensity or prolonged illumination conditions commonly encountered in modern artificial lighting environments.

It is important to distinguish the present mechanistic in vitro findings from the clinical evidence regarding blue light exposure from digital devices. A 2023 Cochrane systematic review of blue-light-filtering spectacle lenses found little or no short-term effect on visual fatigue and little or no clinically meaningful effect on visual performance, while the evidence regarding sleep was uncertain [15]. Importantly, the review identified insufficient randomized clinical evidence to determine effects on macular health. Thus, the current findings should not be interpreted as evidence that routine digital device exposure causes retinal disease in humans. Rather, the present study addresses the cellular oxidative-stress response to controlled blue light irradiation and provides a platform for investigating dose-dependent photobiological responses under defined in vitro conditions.

### 4.3. Discussion of the Effects of Vitamin C on the Generation of ROS in ARPE-19 Cells Under Blue Light Exposure

Vitamin C (ascorbic acid) is a naturally occurring, water-soluble micronutrient that plays essential roles in cellular redox homeostasis, enzymatic reactions, and protection against oxidative stress [53]. In the eye, vitamin C is highly concentrated in ocular tissues, particularly in the aqueous humor and retina, where its levels are substantially higher than those found in plasma [54]. This enrichment reflects its critical function in protecting retinal cells from oxidative damage caused by light exposure and high metabolic activity. As a potent antioxidant, vitamin C directly scavenges a wide range of ROS, acting as a reducing agent by donating electrons to neutralize free radicals, thereby preventing oxidative damage to cellular components such as lipids, proteins, and nucleic acids [55]. Vitamin C suppresses blue-light-induced ROS accumulation in RPE cells by maintaining intracellular redox balance, thereby limiting oxidative damage. Importantly, the antioxidant effects of vitamin C in RPE cells are dose-dependent. At sufficient intracellular concentrations, vitamin C can markedly attenuate or even prevent the increase in ROS induced by blue light exposure, thereby preserving cellular homeostasis under phototoxic conditions. Rózanowska et al. found that in ARPE-19 cells exposed to visible light and a photosensitizer, low-dose ascorbate (350 μM) increased cell viability to ~70% compared with ~42% in untreated controls [56]. In another ARPE-19 oxidative stress model, UV-B irradiation at doses of 20–100 mJ/cm^2^ significantly increased intracellular ROS and reduced cell viability, whereas treatment with ascorbic acid at 500–750 μM markedly improved cell survival under light-induced stress [57]. In an animal model, exposure of isolated rat retinas to 302 nm UVB light for 1 h significantly increased lactate dehydrogenase (LDH) release and disrupted retinal structure, whereas supplementation with 1 mM vitamin C markedly reduced phototoxic damage [58].

The concentrations of vitamin C used in the present study (250 and 500 μM) were selected to examine antioxidant responses over a physiologically relevant range for ocular oxidative-stress studies. Human pharmacokinetic data indicate that oral vitamin C administration produces tightly regulated plasma concentrations, with a mean peak concentration of approximately 135 μM after a 1.25 g oral dose and a modeled peak of approximately 220 μM under repeated high-dose oral administration [59]. Thus, 250 μM is close to the upper physiological plasma range, whereas 500 μM represents a supraphysiological plasma concentration. However, ocular ascorbate concentrations can be substantially higher than plasma concentrations. Human measurements of aqueous humor have reported concentrations in the millimolar range, supporting the relevance of submillimolar vitamin C concentrations for investigating ocular antioxidant responses [60]. The present results in Figure 5 suggest that Vitamin C supplementation noticeably attenuated blue light-induced ROS generation in ARPE-19 cells in a concentration-dependent manner. At 250 μM, vitamin C significantly reduced ROS levels across all luminous intensities, although a modest increase in ROS was still observed as light intensity increased, suggesting partial scavenging of reactive species. In contrast, 500 μM vitamin C effectively suppressed ROS accumulation, maintaining consistently low ROS levels even under high-intensity blue light exposure. Vitamin C exerts strong antioxidant effects by directly scavenging reactive oxygen species and indirectly regenerating other antioxidants, thereby reinforcing intracellular redox defenses and increasing cellular tolerance to phototoxic stress [61]. By effectively limiting blue-light-induced ROS accumulation, vitamin C helps preserve cellular homeostasis and prevent downstream oxidative damage in RPE cells, highlighting its potential as a protective agent against retinal oxidative injury.

### 4.4. Discussion of the Effects of Vitamin E on the Generation of ROS in ARPE-19 Cells Under Blue Light Exposure

Vitamin E is a lipid-soluble antioxidant comprising tocopherols and tocotrienols, with α-tocopherol being the most biologically active form in human tissues. Due to its preferential localization within cellular membranes, vitamin E functions as a chain-breaking antioxidant that suppresses lipid peroxidation by scavenging lipid peroxyl radicals and preserving membrane integrity [62]. This protective role is particularly critical in RPE cells, which are exposed to high oxygen tension, intense light, and photosensitizers such as lipofuscin [63]. Vitamin E has been shown to reduce blue-light- and photo-oxidation-induced ROS accumulation and lipid peroxidation in RPE models, thereby attenuating phototoxic damage [64]. For example, Zinflou et al. found that blue light exposure induced a marked increase in intracellular ROS in ARPE-19 cells, reaching approximately 1.6–1.8-fold above control levels [65]. Pretreatment with 10 μM α-tocopherol (vitamin E) significantly reduced this blue-light-associated ROS elevation, lowering ROS levels to near-baseline values (~1.1–1.2-fold of control), thereby demonstrating its protective antioxidant effect against blue-light-induced oxidative stress. Another study showed that in ARPE-19 cells subjected to photosensitized light exposure, α-tocopherol (10–20 µM) decreased lipid peroxidation by approximately 40–60%, indicating effective suppression of light-induced oxidative stress and ROS propagation within cellular membranes [66]. Blue light irradiation of A2E-loaded ARPE-19 cells caused a marked increase in photo-oxidative stress, whereas α-tocopherol markedly suppressed this response. Specifically, 100 μM vitamin E reduced blue-light-induced A2E epoxide formation by more than ~50%, indicating a substantial attenuation of ROS-driven photo-oxidative reactions in ARPE-19 cells [67]. Wang et al. reported that under high-intensity blue light exposure, intracellular ROS levels increased markedly, whereas vitamin E at 100 μM reduced ROS generation by approximately 40–50% compared with the untreated blue light group, restoring ROS levels toward those of non-irradiated controls [68].

As shown in Figure 6, Vitamin E supplementation markedly attenuated blue-light-induced ROS generation in ARPE-19 cells, demonstrating a strong, concentration-dependent protective effect against phototoxic stress. In contrast to the untreated condition, in which ROS levels increased substantially with increasing luminous intensity, vitamin E effectively suppressed ROS accumulation across all tested light intensities. This protective effect was especially pronounced at higher vitamin E concentrations, where ROS levels remained low even under high-intensity blue light exposure. Upon blue light exposure, ROS-mediated lipid peroxidation is initiated in membrane phospholipids, leading to compromised membrane integrity and downstream cellular dysfunction [69]. Vitamin E interrupts this process by scavenging lipid peroxyl radicals and terminating lipid peroxidation chain reactions, thereby preventing the amplification of oxidative damage within the cell [70]. Notably, the substantial reduction in ROS observed at 250 μM vitamin E suggests that sufficient membrane-associated antioxidant capacity can significantly elevate cellular tolerance to phototoxic stress. These findings underscore the importance of lipid-phase antioxidants in protecting retinal pigment epithelial cells from light-induced oxidative injury and highlight vitamin E as a potent protective agent against blue-light-induced oxidative stress.

Direct comparison of the results obtained with vitamin C (Figure 5) and vitamin E (Figure 6) reveals that both antioxidants effectively attenuate blue-light-induced ROS generation in ARPE-19 cells, albeit through distinct mechanisms and with different response profiles. In the absence of antioxidants, blue light exposure consistently induced a luminous intensity-dependent increase in ROS levels. Supplementation with either vitamin C or vitamin E significantly suppressed this response, demonstrating that both water- and lipid-soluble antioxidants can mitigate phototoxic oxidative stress in retinal pigment epithelial cells. Vitamin C produced a pronounced, dose-dependent suppression of ROS generation, with higher concentrations largely eliminating the light intensity-dependent increase in ROS. This effect is consistent with its function as a water-soluble antioxidant that rapidly scavenges intracellular ROS, particularly under high-intensity blue light exposure. Vitamin E also conferred strong ROS-suppressing effect but through a distinct mechanism. As a lipid-soluble antioxidant localized to cellular membranes, vitamin E effectively limited ROS accumulation even under intense illumination, likely by inhibiting lipid peroxidation and oxidative damage propagation. At higher concentrations, vitamin E maintained uniformly low ROS levels across all light intensities. Together, these results indicate that vitamin C and vitamin E provide complementary protection against blue-light-induced oxidative stress, targeting aqueous and membrane compartments, respectively, and underscore the value of multi-compartment antioxidant defense in retinal pigment epithelial cells.

### 4.5. Discussion of the Effects of Uric Acid on the Generation of ROS in ARPE-19 Cells Under Blue Light Exposure

As shown in Figure 7b, UA treatment significantly reduced ROS generation at all tested light intensities. At 0.05 mg/mL UA, ROS levels ranged from 24.2 to 29.0, representing an approximately 17–29% reduction compared with the corresponding control groups without UA. A stronger suppression was observed at 0.1 mg/mL UA, where ROS levels decreased to 15.4–25.2, corresponding to reductions of roughly 35–48%. These results demonstrate a clear concentration-dependent antioxidant effect of UA in ARPE-19 cells. UA is known to function as a potent endogenous antioxidant in biological systems by scavenging reactive oxygen and nitrogen species and by contributing significantly to the antioxidant capacity of human plasma [71,72]. Its ability to neutralize free radicals may therefore explain the reduced ROS signals observed in the presence of UA. As reported, UA serves as a primary defense against oxidative stress in the human internal environment, accounting for approximately 60% of the total antioxidant capacity in plasma [73]. This protective effect is particularly evident in models of cerebral ischemia–reperfusion, where concentrations of 300 μM (~0.05 mg/mL) significantly reduce ROS generation and enhance neuronal cell viability [74]. Furthermore, UA demonstrates high specificity in neutralizing specialized reactive species; for instance, it effectively blocks peroxynitrite-mediated nitrosylation and, at levels of 0.6 mM (~0.1 mg/mL), has been shown to provide critical protection against UV-induced oxidative damage in biological models [75].

The qualitative fluorescence images in Figure 7a further support the quantitative measurements. DCFDA fluorescence intensity visibly increased with increasing illumination intensity in the absence of UA, indicating enhanced ROS accumulation under stronger blue light exposure. In contrast, cells treated with UA displayed weaker fluorescence signals, particularly at the higher UA concentration. Importantly, DIC images showed no obvious morphological deterioration across the tested conditions, suggesting that the differences in fluorescence primarily reflect changes in intracellular ROS levels rather than large-scale structural damage to the cells.

The present results indicate that UA effectively suppresses this oxidative stress in a concentration-dependent manner, suggesting that it may act as a protective antioxidant against blue-light-induced retinal damage. These findings provide additional insight into the role of antioxidants in mitigating light-induced oxidative stress in RPE cells and may have implications for the development of strategies aimed at protecting the retina from phototoxic injury associated with modern light-emitting devices. Future studies investigating downstream cellular responses, including mitochondrial function, lipid peroxidation, and apoptosis signaling pathways, would further clarify the mechanisms underlying the protective effects of UA in retinal cells.

### 4.6. Further Studies

The present study provides evidence of blue-light-induced oxidative stress in ARPE-19 cells through intracellular ROS measurements, together with complementary cell-viability assessments. Blue light exposure increased intracellular ROS levels in an illuminance-dependent manner, whereas vitamin C, vitamin E, and uric acid attenuated ROS accumulation in a concentration-dependent manner. Importantly, 2 h exposure to 2500-lux blue light did not produce a detectable reduction in cell viability in the presence of the tested antioxidants. These findings suggest that the observed ROS changes were not accompanied by detectable cytotoxicity under the tested conditions. Several limitations remain. First, the blue light exposure conditions used in this study should be considered in relation to typical digital-device exposure. The cells were exposed to blue light for 2 h, with irradiances ranging from 0.61 to 5.65 mW/cm^2^ and corresponding energy doses of 4.4 to 40.7 J/cm^2^. These doses are substantially higher than those reported for commonly used digital devices, which vary considerably with device type, screen brightness, and viewing distance [76]. Therefore, the exposure conditions in the present study were intended to establish a controlled and measurable in vitro dose–response range rather than to directly simulate typical digital-device use. Future studies should investigate lower irradiances and longer exposure durations that more closely reflect real-world exposure conditions. Second, H_2_DCFDA provides an overall measure of intracellular ROS but does not identify specific ROS or directly demonstrate downstream oxidative damage. Future studies should therefore include measurements of lipid, protein, and DNA oxidation, as well as endogenous antioxidant responses. In addition, apoptosis, necrosis, and mitochondrial dysfunction were not directly evaluated. Assays of apoptosis, mitochondrial membrane potential, respiratory function, and immunocytochemical analysis of relevant oxidative-stress, mitochondrial, and cell-death markers would provide further insight into the biological consequences of blue light exposure. Finally, the present experiments were performed using an in vitro ARPE-19 cell model, which cannot fully reproduce the complexity of the intact human retina. The experimental light doses also should not be considered direct equivalents of typical digital-device exposure. Future studies using more physiologically relevant retinal models, broader light-dose and exposure-duration ranges, and multiple biological endpoints will help further validate the applicability of this platform to retinal photobiological and phototoxicity studies.

## 5. Conclusions

In this study, a microfluidic platform capable of simultaneously generating gradients of blue light intensity and antioxidant concentration was successfully developed and applied to evaluate oxidative stress responses in ARPE-19 RPE cells. By integrating a Christmas-tree microfluidic gradient generator with a light-attenuation module using Congo red, the device produced well-defined and reproducible blue light illuminance levels while enabling controlled delivery of chemical treatments within the same experimental system. This design allowed the simultaneous assessment of 15 different combinations of light exposure and antioxidant concentrations in a single experiment, significantly improving experimental efficiency while reducing reagent consumption. Experimental results demonstrated a clear blue-light-intensity-dependent increase in intracellular ROS generation in ARPE-19 cells. Conversely, the antioxidants vitamin C, vitamin E, and uric acid effectively suppressed ROS accumulation, with higher antioxidant concentrations providing stronger protection against blue-light-induced oxidative stress. These findings support the utility of the platform for investigating light-induced oxidative stress and antioxidant responses in retinal pigment epithelial cells. Further studies incorporating mitochondrial function, apoptosis, and immunocytochemical endpoints will be required to determine whether the observed reduction in ROS translates into broader cellular protection. Overall, the microfluidic platform developed in this work provides a controllable and physiologically relevant in vitro system for investigating photo-oxidative stress in retinal cells. The ability to integrate optical and chemical gradients within a single device offers a powerful tool for mechanistic studies of retinal damage and for high-throughput screening of potential protective compounds. This approach may facilitate future investigations into therapeutic strategies aimed at mitigating blue-light-induced retinal injury and could contribute to the development of preventive interventions for retinal degenerative diseases.

## Figures and Tables

**Table 1:** Calculating the absorbance of Congo red to blue light.

**Congo red concentration. (*c* in mg/mL).**	0	0.3125	1.25	2.1875	2.5
**Mean measured illuminance (lux)**	2557 ± 82	2453 ± 102	1100 ± 82	510 ± 134	277 ± 76
**Estimated illuminance experienced by cells (lux)**	2260 ± 76	2169 ± 90	973 ± 73	451 ± 118	245 ± 67
**Irradiance (mW/cm^2^)**	5.65 ± 0.19	5.42 ± 0.23	2.43 ± 0.18	1.13 ± 0.30	0.61 ± 0.17
**Energy dose (J/cm^2^)**	40.7 ± 1.4	39.0 ± 1.6	17.5 ± 1.3	8.1 ± 2.1	4.4 ± 1.2
**Normalized transmittance (*T*)**	1.00 ± 0.03	0.96 ± 0.04	0.43 ± 0.03	0.20 ± 0.05	0.11 ± 0.03
		*A* = −log*T*			
**Absorbance (*A*)**	0.000 ± 0.015	0.018 ± 0.018	0.367 ± 0.032	0.717 ± 0.125	0.981 ± 0.116
**Equation**	*A* = 0.3534*c*				
**R^2^**			0.964		

## Data Availability

Data will be made available upon reasonable request.

## Supplementary Materials

The following supporting information can be downloaded at: https://www.mdpi.com/article/10.3390/bios16090516/s1, Figure S1: Cell viability of ARPE-19 cells under dark and blue-light conditions in the presence of different antioxidant concentrations; Figure S2: ROS intensity of ARPE-19 cells under dark and blue-light conditions in the presence of different antioxidant concentrations.
