# Supplementary material for: A Microfluidic Gradient Platform for High-Throughput Evaluation of Blue-Light-Induced Oxidative Stress and Antioxidant Protection in Retinal Pigment Epithelial Cells
Source: Biosensors (Basel). 2026 Sep 12;16(9):516. doi: 10.3390/bios16090516 (PMC13604456; doi:10.3390/bios16090516)
Supplement: Supplementary file 1 [file biosensors-16-00516-s001.zip › biosensors-4476663-supplementary.pdf]

Supplementary files of "**A microfluidic gradient platform for high-throughput evaluation of blue-light-induced oxidative stress and antioxidant protection in retinal pigment epithelial cells**"

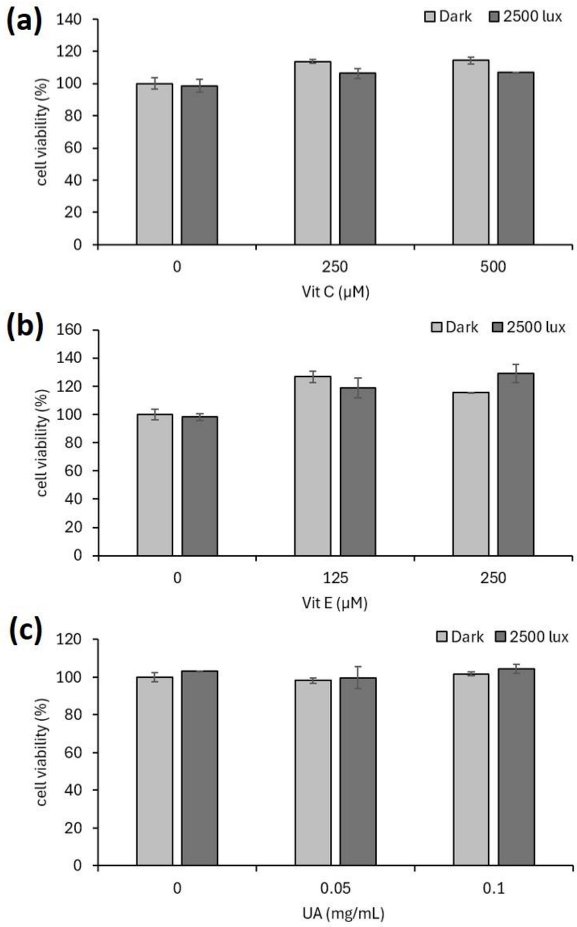

Figure S1. Cell viability of ARPE-19 cells under dark and blue-light conditions in the presence of different antioxidant concentrations. (a) Vitamin C (VC), (b) vitamin E (VE), and (c) uric acid (UA). Cell viability was measured under dark conditions (Dark) and after exposure to 2500-lux blue light (2500 lux), and then normalized to 100% under dark conditions at a concentration of 0 for each antioxidant. The *x*-axis indicates the concentration of each antioxidant.

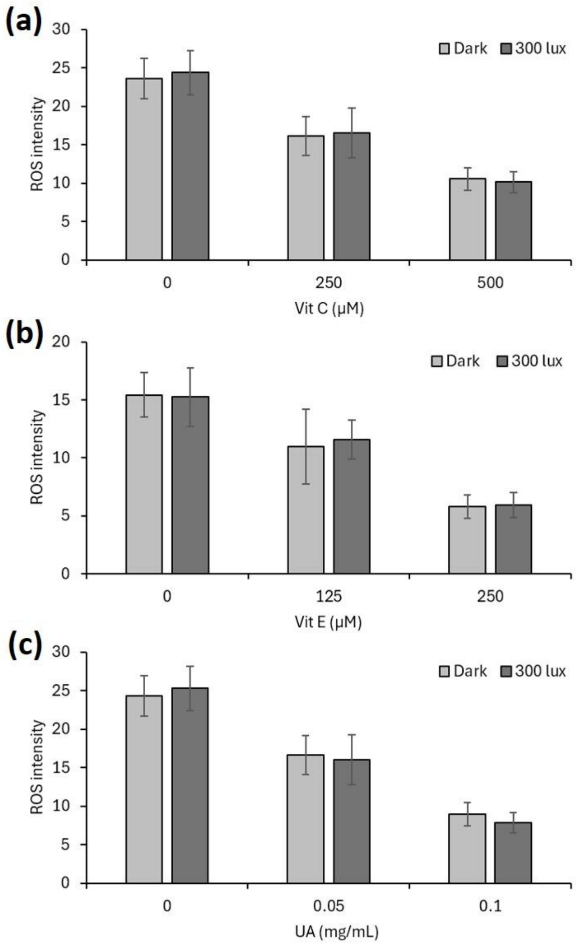

Figure S2. ROS intensity of ARPE-19 cells under dark and blue-light conditions in the presence of different antioxidant concentrations. (a) Vitamin C (VC), (b) vitamin E (VE), and (c) uric acid (UA). ROS intensity was measured under dark conditions (Dark) and after exposure to 300-lux blue light (300 lux). The *x*-axis indicates the concentration of each antioxidant.
